# Circulating lipids in men with type 2 diabetes following 3 days on a carbohydrate‐free diet versus 3 days of fasting

**DOI:** 10.14814/phy2.14569

**Published:** 2020-10-08

**Authors:** Frank Q. Nuttall, Rami M. Almokayyad, Mary C. Gannon

**Affiliations:** ^1^ Section of Endocrinology, Metabolism & Nutrition, and the Metabolic Research Laboratory Minneapolis VA Health Care System University of Minnesota Minneapolis MN USA; ^2^ Department of Medicine University of Minnesota Minneapolis MN USA; ^3^ Department of Food Science & Nutrition University of Minnesota St Paul MN USA; ^4^ Present address: Park Nicollet Health Care System St. Louis Park MN USA

**Keywords:** carbohydrate‐free diet, high fat diet, non‐esterified fatty acids, triacylglycerol

## Abstract

**Objective:**

We have been interested in determining the effects of dietary changes on fuel metabolism and regulation in men with type 2 diabetes mellitus (T2DM). In this study, the changes in 24‐hr circulating lipid profiles were determined when the major fuel source was endogenous versus exogenous fat.

**Methods:**

Seven males with T2DM were randomized in a crossover design with a 4‐week washout period. A standard mixed (control) diet (30%fat:15%protein:55%carbohydrate) was provided initially. Subsequently, a 72‐hr (3‐day) fast, or a high fat (85%), 15% protein, essentially carbohydrate‐free (CHO‐free) diet was provided for 72 hr. Triacylglycerol (TAG), non‐esterified fatty acids (NEFA), β‐hydroxybutyrate (bHB), and insulin‐like growth factor‐binding protein‐1 (IGFBP‐1) profiles were determined during the last 24 hr of intervention, as well as during the control diet.

**Results:**

Regardless of the amount of dietary fat (30% vs 85%) and differences in 24‐hr profiles, TAG, NEFA, and bHB all returned to the previous basal concentrations within 24 hr. TAGs and NEFAs changed only modestly with fasting; bHB was elevated and increasing. The IGFBP‐1 profile was essentially unchanged with either diet but increased with fasting.

**Conclusion:**

A CHO‐free diet resulted in a large increase in TAG and NEFA versus the control diet; however, both were cleared by the following morning. A negative NEFA profile occurred with the control diet. Thus, mechanisms are present to restore lipid concentrations to their original AM concentrations daily. Fasting resulted in stable concentrations, except for a continuing increase in bHB. Glucose and insulin, common fuel regulators, could not explain the results.

## Graphical abstract

The 24‐hr response profiles for TAG, NEFA, bHB, and IGFBP‐1 were obtained when ingesting a mixed control diet before, and during the last 48–72 hr when ingesting a CHO‐free diet or fasting in male subjects with untreated type 2 diabetes. Data indicate that subjects are still able to absorb, process, utilize, and if necessary, store large amounts of ingested fats adequately, as well as adjust adequately to short‐term starvation.

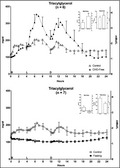

## 1 INTRODUCTION

Humans as well as other animals must adjust their metabolic response to variations in fuel supply. In humans, the major adjustment is a quantitative adaptation to the relative amounts of carbohydrate‐ (glucose) and lipid‐derived fuel available for oxidization. Glucose is the preferred general fuel. However, when not available in adequate amounts, fat‐derived fuel must be used (Whitley et al., 1997). Thus, there is a reciprocal relationship in their utilization.

We have been interested in understanding the metabolic consequences of changes in diet in people with T2DM. We previously reported the 24‐hr glucose, insulin, and glucagon responses to a 72‐hr fast and to a 72‐hr macronutrient sufficient, no carbohydrate (CHO), high‐fat diet (CHO‐free diet) in men with type 2 diabetes (Nuttall, Almokayyad, & Gannon, 2015). We also have reported the 24‐hr responses of plasma ghrelin and leptin (Nuttall, Almokayyad, & Gannon, 2016) and well as the quantitative effect on general fuel metabolism in the same subjects (Nuttall, Almokayyad, & Gannon, 2018).

In this publication, we report the circulating fat‐derived products, that is, triacylglycerol (TAGs), non‐esterified fatty acids (NEFAs), and β‐hydroxybutyrate (bHB), in the same subjects. In addition, although not lipid related, IGF Binding Protein‐3 (IGFBP‐3) as well as free insulin‐like growth factor I (IGF‐I) data were obtained. The IGF‐1 is known to be controlled by insulin (Gannon and Nuttall, 2011). In addition, the insulin data reported previously (Nuttall et al., 2015) has been reformatted and included for discussion purposes.

To the best of our knowledge 24‐hr comparative lipid data have not been published. Indeed, 24‐hr profile data are uncommon due to the cost and difficulty in doing such studies. Also, the great majority of publications related to lipid utilization have been in nondiabetic subjects, have been of relatively short duration and have been designed to perturb a metabolic system in order to better understand mechanisms regulating that metabolic pathway. To the best of our knowledge the only 24‐hr lipid‐based data (fatty acids) in people with T2DM was published in1988 by Reaven, Hollenbeck, Jeng, Wu, & Chen (1988).

## 2 MATERIALS AND METHODS

### 2.1 Subjects

Seven male subjects with type 2 diabetes were studied in a clinical research unit (Special Diagnostic and Treatment Unit, SDTU). The diabetes in one subject was untreated; three subjects had been receiving metformin. Three subjects had been receiving glipizide. After consultation with the patient, and the approval of the primary care provider, these medications were discontinued for 24 or more days before the study as well as during the entire study. Thus, during the study, the patients’ diabetes was “untreated.” Patient characteristics have been published previously (Nuttall et al., 2015). Briefly, the mean age was 60 years (range 49–72), mean weight was 97 ± 6 kg (range 81–130), mean BMI 31 ± 2 kg/m^2^ (range 27–38). The previous dates of diabetes diagnosis varied from 3 to 18 years; six of the seven subjects were taking a statin‐type medication which was continued during the study. (Please see ref (Nuttall et al. 2015) for details.) Written informed consent was obtained from all subjects, and the study was approved by the Department of Veterans Affairs Medical Center Internal Review Board (IRB). The study was registered with ClinicalTrials.gov, Identifier: NCT01469104.

### 2.2 Study design

A randomized crossover study design with a 4‐week washout period was used. The subjects initially ingested a standard mixed diet (control) consisting of 55% CHO, 15% protein, 30% fat (day 1), then either a high fat, essentially carbohydrate‐free (CHO‐free) diet consisting of <3% carbohydrate, 15% protein, ~85% fat (detailed in Nuttall et al., 2018), or fasted for 72 hr (days 2–4). All subjects participated in both study arms, that is, fasting for 72 hr and ingesting a CHO‐free diet for 72 hr. Throughout the manuscript, the standard, nutrient‐sufficient, mixed diet of 55% carbohydrate 30% fat, 15% protein is referred to as the control diet.

Our subjects were self‐reported sedentary individuals, and they were requested to avoid any strenuous activities on the days before the study. The day before admission (day 0), each subject was provided with a standardized dinner (55% carbohydrate, 15% protein, and 30% fat), to be ingested at home at 1800 hr.

Each subject reported to the SDTU at 0700 hr. An indwelling IV catheter was inserted into an antecubital vein and blood samples were obtained at 0730, 0745, and 0800 hr for baseline determinations. Subjects received the control diet, breakfast, lunch, and dinner at 0800, 1200, and 1800 hr, respectively. Blood samples were obtained before and every 15 min after each meal for the first hr, every 30 min for the second and third hr and hourly thereafter until the next meal or until 0800 the next morning. This represents day 1.

On day 2, subjects were asked to starve for 72 hr or were provided with the CHO‐free meals at 0800, 1200, and 1800 hr each day during the 72 hr of the study. This represents days 2–4. The total food energy distribution was breakfast 32%, lunch 40%, and dinner 28%. It was considered ideal to have each meal contain the same total food energy (i.e., to consider circadian effects). This was not possible due to having diet contents acceptable to the subjects.

Ingestion of water was encouraged. Black coffee, tea without sugar or cream, and calorie‐free beverages were allowed, but not encouraged. Activity was limited to quiet diversions such as reading or watching TV. Blood samples were obtained during last 24 hr of the 72‐hr intervention (i.e., day 4), with the same time intervals as the first day. Urine was collected during the 24 hr of day 1, and the last 24 hr of the 72‐hr intervention (day 4). The subjects were under continuous supervision and were not allowed to leave the research unit.

Plasma or serum was analyzed for triacylglycerol (TAG), non‐esterified fatty acids (NEFA), β‐hydroxybutyrate (bHB), and insulin‐like growth factor binding protein 1 (IGFBP‐1) over two 24‐hr periods (days 1 and 4). Some blood samples were not available during the overnight hours for one subject while ingesting the CHO‐free diet. Therefore, that complete data set, with the corresponding control data, is presented for six subjects. In this report, we separated the control data for comparison in each arm of the study to provide a true pre‐ and post‐result. In addition, only overnight fasting (0800 hr) blood samples were analyzed for total‐ VLDL‐ LDL‐ HDL‐ and non‐HDL‐cholesterol, free insulin‐like growth factor‐I (IGF‐I), and insulin‐like growth factor binding protein 3 (IGFBP‐3) on days 1 and 4 (Table 1).

NOTE: The glucose and insulin results were published previously (Nuttall et al., 2015) and are included in this manuscript (Figures 5 and 6) for clarity in the discussion. In the previous publication the data for the two control‐diet days were combined, since there was little difference in the responses. In this report, the individual control day data are separated to conform to the current presentation. Thus, the glucose and insulin figures from an earlier publication are not identical to those presented here.

### 2.3 Assays

Serum free TAG, total cholesterol, and HDL‐cholesterol were determined by an automated method on an Abbott Architect ci 8200 analyzer; LDL‐cholesterol was calculated by the Friedwald formula. The VLDL‐cholesterol concentration was estimated by dividing the triglyceride concentration (in mmol/L) by 2.2. NEFAs were determined with a kit from Wako (Wako Chemicals USA Inc., Richmond, VA; now Wako Diagnostics, division of Fujifilm) using oleic acid as a standard. IGF‐I, IGFBP‐1, and IGFBP‐3 were determined using ELISA kits manufactured by Diagnostic Systems Laboratory (DSL), Webster, TX (now part of Beckman Coulter); bHB was determined with the Stanbo LiquiColor Method, Stanbio Laboratory, Boerne, TX.

Dietary content of protein, fat and CHO was estimated using diet analysis modules “Nutritionist Pro” (Axxya Systems LLC, Stafford, TX) and “VistA” (Veterans Health Information Systems and Architecture; Department of Veterans Affairs, Veterans Health Administration, US Government). Both are based on United States Department of Agriculture (USDA) databases.

### 2.4 Statistics

The area responses were calculated with a computer program based on the trapezoid rule (Gannon, 1991). Net area refers to the area in relation to the fasting (0800 hr) baseline. Total area refers to the area above zero. Statistics were determined using Student's paired *t*‐test for Area data. For comparisons between diets or between time points in control versus intervention in Time Course data, a two‐way repeated measures ANOVA was used with a Bonferroni correction (GraphPad Prism, San Diego, CA, version 8.4.3 for Mac.) A *p*‐value equal to or less than .05 was the criterion for significance. Data are presented as the mean ± *SEM*.

## 3 RESULTS

### 3.1 Plasma triacylglycerol (TAG)

**FIGURE 1:**
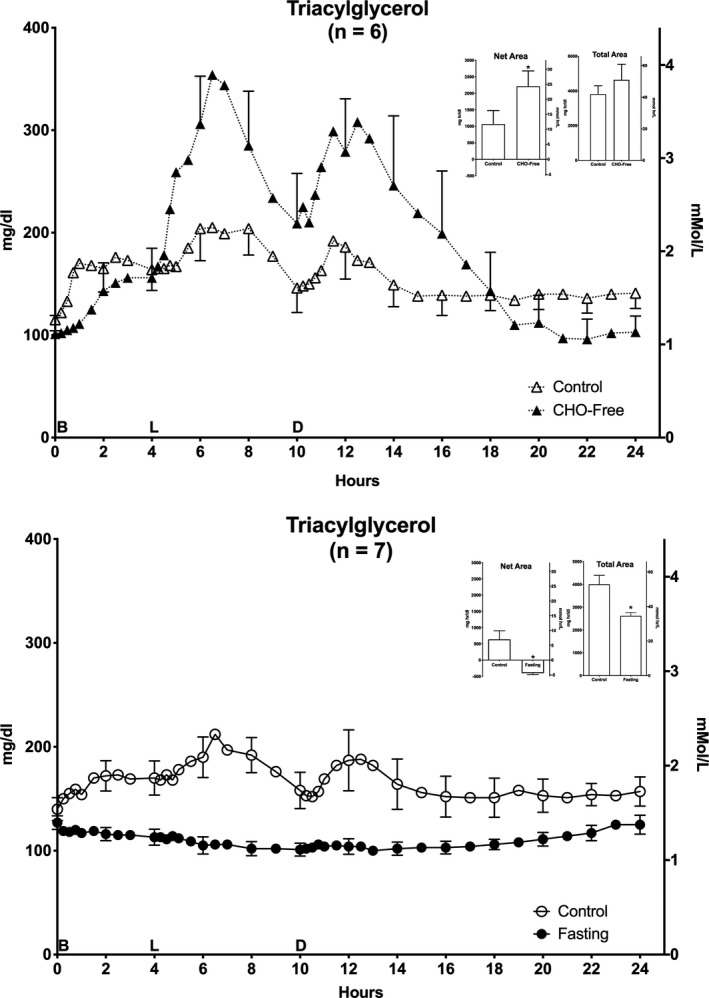
Plasma Triacylglycerol (TAG). Twenty‐four hour triacylglycerol responses. Top: (*n* = 6) Mean ± standard error of the mean (*SEM*) while ingesting the control diet (open triangles) and for the last 24 hr (hours 48–72) of ingesting CHO‐free diet (closed triangles). Bottom: (*n* = 7) Mean ± *SEM* while ingesting the control diet (open circles) and the last 24 hr (hours 48–72) of fasting (closed circles). X‐axis: Time in hours. 0 = 0800 hr. B, L, D indicate breakfast, lunch and dinner mealtimes. Y Axis: Left – concentration in Imperial units, Right – concentration in Scientific Units. Inserts: Net Area represents the mean ± *SEM* integrated 24‐hr area response, using the overnight fasting concentration as baseline. Total Area represents the mean ± *SEM* integrated 24‐hr area response, using zero as baseline. S = Standard mixed diet (control), C = CHO‐free diet, *F* = Fasting. Statistics were done using Student's *t* test for paired variates. *p < .05

Following ingestion of the control diet (day 1) for the CHO‐free arm of the study, the initial (0800 hr) TAG concentration was 1.3 ± 0.1 mmol/L (Figure 1 – top). The TAG concentration increased and remained elevated until hr 14 with a further modest increase after each meal. The final concentration was 1.6 ± 0.2 mmol/L, that is, it was still modestly elevated. The maximum occurred at ~hr 6.5 (2.3 ± 0.3 mmol/L).

When ingesting the CHO‐free diet (day 4), the initial (0800 hr) TAG concentration was 1.1 ± 0.2 mmol/L, that is, similar to when the control diet was consumed. The concentration then increased markedly. The profiles were similar. The maximum also occurred at hr 6.5 (3.9 ± 0.7 mmol/L). Although the increase was much greater, it subsequently returned to the baseline (1.1 ± 0.2 mmol/L) by hr 24 (0800 hr the following morning).

Prior to the 3‐day fast, both the initial (0800 hr) TAG concentration (1.5 ± 0.1 mmol/L) for the control diet (day 1) (Figure 1 – bottom) as well as the 24‐hr profile were very similar. This was in spite of the fact that the data were obtained ~5 weeks apart. On the three occasions in which food was ingested, the temporal patterns were grossly similar, though greatly exaggerated when the CHO‐free diet was ingested. On the last day of the fast, the initial (0800 hr) TAG concentration was 1.4 ± 0.1 mmol/L. There was a very modest decrease in TAG concentration during the day. The final concentration at the end of the fast was 1.4 ± 0.1 mmol/L, that is, it had returned to the initial value.

#### 3.1.1 Areas

(Top insert) The 24‐hr integrated net TAG area response (left) when on the control diet (day 1), was 12 ± 5 mmol·hr/L. It was 24 ± 6 mmol·hr/L for the CHO‐free diet, a 108% increase (*p* < .05). The total area responses (right) were 42.0 ± 5.3, and 51.1 ± 7.6 mmol·hr/L (22% increase), respectively. The difference was not statistically significant (*p* = .15) and likely is due to the modestly reduced clearance during the last 8 hr of the 24 hr control data.

(Bottom insert) For the control diet prior to fasting (day 1), the integrated net TAG area response (left) was 7.0 ± 2.9 mmol·hr/L. Due to fasting (day 4), it was −4.4 ± 0.4 mmol·hr/L, a 163% decrease (*p* = .004) (Figure 1). The respective total area responses (right) were 44.0 ± 4.3 and 28.8 ± 1.6 mmol·hr/L, a 35% decrease (*p* = .004). Thus, the area data indicate a significant increase in the net integrated 24‐hr concentration with the CHO‐free diet compared to its respective control diet, while with fasting both the net and total responses were decreased compared to the respective control diet.

### 3.2 Non‐esterified fatty acids (NEFA)

**FIGURE 2:**
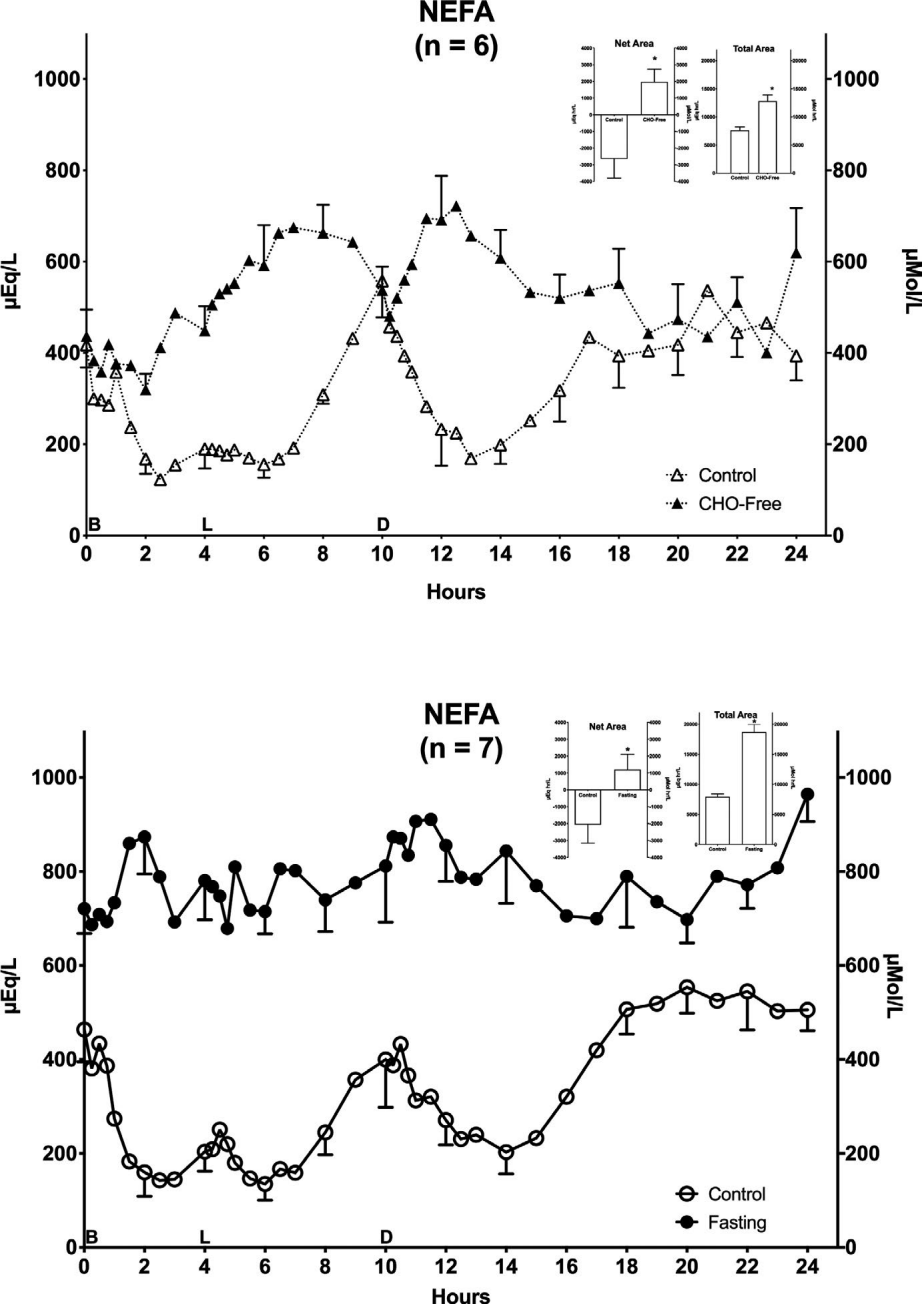
Plasma Non‐Esterified Fatty Acids (NEFA). Twenty‐four hour NEFA responses. Top: (*n* = 6) Mean ± standard error of the mean (*SEM*) while ingesting the control diet (open triangles) and for the last 24 hr (hours 48–72) of ingesting CHO‐free diet (closed triangles). Bottom: (*n* = 7) Mean ± *SEM* while ingesting the control diet (open circles) and the last 24 hr (hours 48–72) of fasting (closed circles). X‐axis: Time in hours. 0 = 0800 hr. B, L, D indicate breakfast, lunch and dinner mealtimes. Y Axis: Left – concentration in Imperial units, Right – concentration in Scientific Units. Inserts: Net Area represents the mean ± *SEM* integrated 24‐hr area response, using the overnight fasting concentration as baseline. Total Area represents the mean ± *SEM* integrated 24‐hr area response, using zero as baseline. S = Standard mixed diet (control), C = CHO‐free diet, F = Fasting. Statistics were done using Student's *t* test for paired variates. *p < .05

Prior to ingestion of the control diet (day 1), the initial NEFA concentration (0800 hr) was 418 ± 50 µmol/L (Figure 2 – top). The NEFA concentration then rapidly decreased and reached a nadir at hour 2.5, remained low until hour 6 when it began to rise rapidly back to the overnight fasting value. It then again rapidly decreased reaching a second nadir at hour 13. It gradually returned to the previous overnight fasting value (394 ± 54 µmol/L).

When the CHO‐free diet was ingested, the initial concentration on day 4 (0800 hr) was 437 ± 58 µmol/L (Figure 2 – top). After a transient decrease (hr 2) and in contrast with the control diet, the NEFA concentration increased throughout the day with only a transient decrease prior to the dinner meal (hr 10). It then decreased to the same concentration as when subjects ingested the control meal. At the end of hr 23 it had returned to the initial overnight fasting value (401 ± 65 µmol/L). However, for an unexplained reason, the last hr 24 concentration was considerably elevated (mean 620 ± 97 µmol/L).

The NEFA results while on the control diet for the fasting arm (Figure 2 ‐ bottom) were very similar to those of the control diet prior to ingestion of the CHO‐free diet, except that at hr 24 the concentration had returned to near the initial value (506 ± 45 µmol/L). The general responses with the control diets was a decrease after meals, a modest increase before lunch, a greater increase before dinner, and an increase beginning ~4 hr after dinner. In contrast, with the CHO‐free meals, there were increases in NEFA concentrations after the meals.

On the last day of fasting (day 4), the initial (0800 hr) NEFA concentration had increased by approximately 55% (from 464 ± 69 with the control diet to 721 ± 53 µmol/L with fasting). It remained increased and more or less stable but with an overall modest increase and a maximum at hr 11. At hr 24, that is, the next day 0800‐hr value, it was further increased to 965 ± 59 µmol/L (*p* = .01). Whether this represents a further upward trend remains to be determined but is likely. Thus, in contrast to the large meal‐related increases in concentration following the CHO‐free diet, with fasting the diurnal concentration was markedly attenuated.

#### 3.2.1 Areas

(Top insert) The 24‐hr integrated net NEFA area responses (left) were −2645 ± 1,147 and +1,972 ± 767 µmol·hr/L for the control diet and the CHO‐free diet, respectively (175% increase) (*p* = .0003). The total area responses (right) were 7,602 ± 612 and 12,849 ± 1,063 µmol·hr/L, respectively, for the control diet and the CHO‐free diet (69% increase) (*p* = .0005).

(Bottom insert) With the control diet and fasting, net NEFA area responses (left) were −2069 ± 1,098 and +1,203 ± 906 µmol·hr/L, respectively (158% increase) (*p* = .0181). The total area responses (right) were 7,956 ± 457 and 18,696 ± 1,275 µmol·hr/L, respectively (135% increase) (*p* = .0001).

Thus, overall, both with a CHO‐free diet and with fasting, the net and total area responses were significantly increased compared to their respective controls.

### 3.3 β‐Hydroxybutyrate (bHB)

**FIGURE 3:**
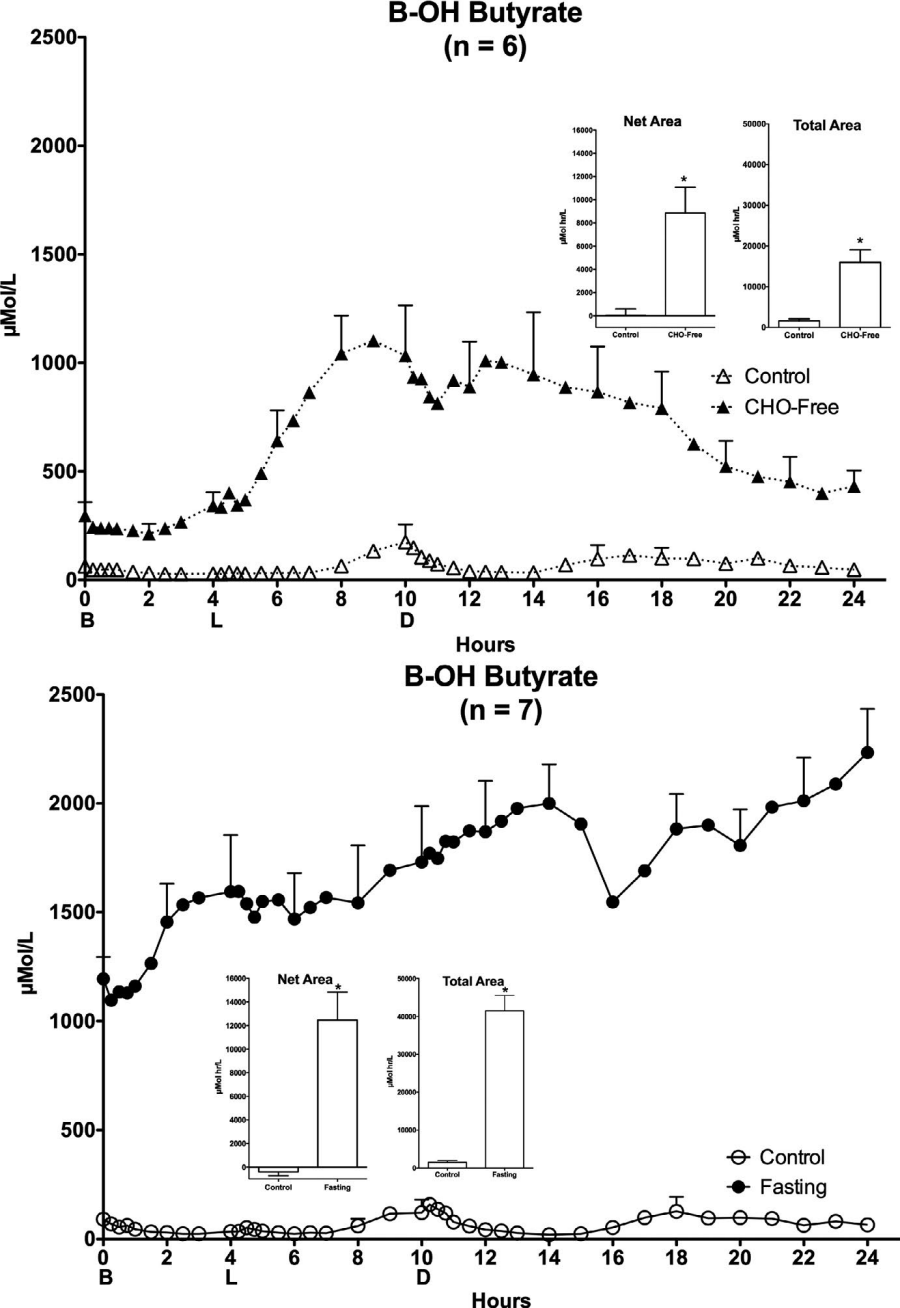
Plasma β‐Hydroxybutyrate (bHB). Twenty‐four hour bHB responses. Top: (*n* = 6) Mean ± standard error of the mean (*SEM*) while ingesting the control diet (open triangles) and for the last 24 hr (hours 48–72) of ingesting CHO‐free diet (closed triangles). Bottom: (*n* = 7) Mean ± *SEM* while ingesting the control diet (open circles) and the last 24 hr (hours 48–72) of fasting (closed circles). X‐axis: Time in hours. 0 = 0800 hr. B, L, D indicate breakfast, lunch and dinner mealtimes. Y axis Left, concentration in Scientific Units. Inserts: Net Area represents the mean ± *SEM* integrated 24‐hr area response, using the overnight fasting concentration as baseline. Total Area represents the mean ± *SEM* integrated 24‐hr area response, using zero as baseline. S = Standard mixed diet (control), C = CHO‐free diet, *F* = Fasting. Statistics were done using Student's *t* test for paired variates. *p < .05

The control diet initial mean fasting bHB concentration was 64 ± 11 µmol/L (Figure 3‐top). It promptly decreased by about 50% and remained low until hr 8 when it rather abruptly increased and reached a maximum at hr 10. It then decreased and remained low until hr 16 when it increased back to the initial mean concentration (65 ± 11 µmol/L).

The CHO‐free diet resulted in an increase in the overnight fasting (0800 hr) bHB concentration to 297 ± 61 µmol/L, that is, ~5‐fold increase compared to the control diet. Subsequently, it remained little changed until hr 4 when it increased rapidly, reached a maximum of 1,103 ± 180 µmol/L at hr 9, increased again and then gradually decreased. It did not completely (433 ± 70 µmol/L) return to the previous overnight fasting value.

The control diet associated with the 24‐hr fasting arm of the study resulted in an initial bHB concentration similar to that of the CHO‐free arm of the study and the dynamic changes throughout the day also were very similar.

The initial overnight bHB concentration on the last day of fasting was greatly elevated (1,194 ± 101 µmol/L) (Figure 3 – bottom) compared to the control diet (91 µmol/L), that is, a 1,212% increase. It increased further until hr 14, decreased transiently and then continued to increase. The final concentration was 2,233 ± 200 µmol/L (*p* = .001), that is, an 87% increase over the initial fasting concentration even though the corresponding NEFA concentration increased only 34%.

#### 3.3.1 Areas

(Top insert) The mean net 24‐hr area response (left) was 71 ± 526 µmol·hr/L, for the control diet and 8,862 ± 1,860 µmol·hr/L for the CHO‐free diet, respectively (12,382% increase) (*p* .01). The total area responses (right) were 1614 ± 507 and 15,999 ± 3,073 µmol·hr/L for the control diet and CHO‐free diet, respectively (891% increase) (*p* = .006).

(Bottom insert) For the control diet and fasting, the mean net 24‐hr area response (left) was −395 ± 325 and 12,471 ± 2,369 µmol·hr/L, respectively, (~3,250% increase) (*p* = .001). The total area responses (right) were 1507 ± 445 for the control diet and 41,486 ± 4,063 µmol·hr/L with fasting (2,653% increase) (*p* = .0001).

### 3.4 Other lipid data

The 0800‐hr total cholesterol, LDL‐cholesterol, HDL‐cholesterol, VLDL‐cholesterol, and Non‐HDL‐cholesterol concentrations were essentially unchanged (Table 1).

**TABLE 1:** Plasma/serum 0800 hr data

Plasma/Serum	*N* = 7					
	Meals					
	Pre‐ CHO free Day 1	CHO‐free Day 4	∆	Pre fasting Day 1	Fasting Day 4	∆
Total cholesterol (mmol/L)	3.9 ± 0.36	3.9 ± 0.41	0	4.0 ± 0.38	4.0 ± 0.44	0
LDL‐cholesterol (mmol/L)	2.4 ± 0.33	2.5 ± 0.38	0.1	2.3 ± 0.03	2.5 ± 0.44	0.2
HDL‐cholesterol (mmol/L)	0.9 ± 0.08	0.9 ± 0.05	0	1.0 ± 0.10	0.9 ± 0.10	−0.1
VLDL‐cholesterol (mmol/L)	0.6 ± 0.05	0.5 ± 0.08	−0.1	0.7 ± 0.10	0.6 ± 0.03	−0.1
Non HDL‐cholesterol (mmol/L)	3.0 ± 0.33	3.0 ± 0.38	0	3.0 ± 0.38	3.1 ± 0.44	0.1
IGF‐I (nmol/L)	10.5 ± 4.76	9.2 ± 0.64*	−1.3	11.1 ± 0.18	10.5 ± 4.73	−0.6
IGFBP−3 (µg/L)	1,297 ± 187	1,226 ± 167	−71	1,241 ± 184	1,287 ± 176	46

Note

∆, post minus pre value.

CHO, carbohydrate; HDL, high‐density lipoprotein; IGFBP‐3, Insulin‐Like Growth Factor Binding Protein‐3; IGF‐I, Insulin‐like growth factor I; LDL, low‐density lipoprotein; VLDL, very low density lipoprotein.

* *p* < 0.05 using Student's paired *t* test.

### 3.5 IGFBP‐1

**FIGURE 4:**
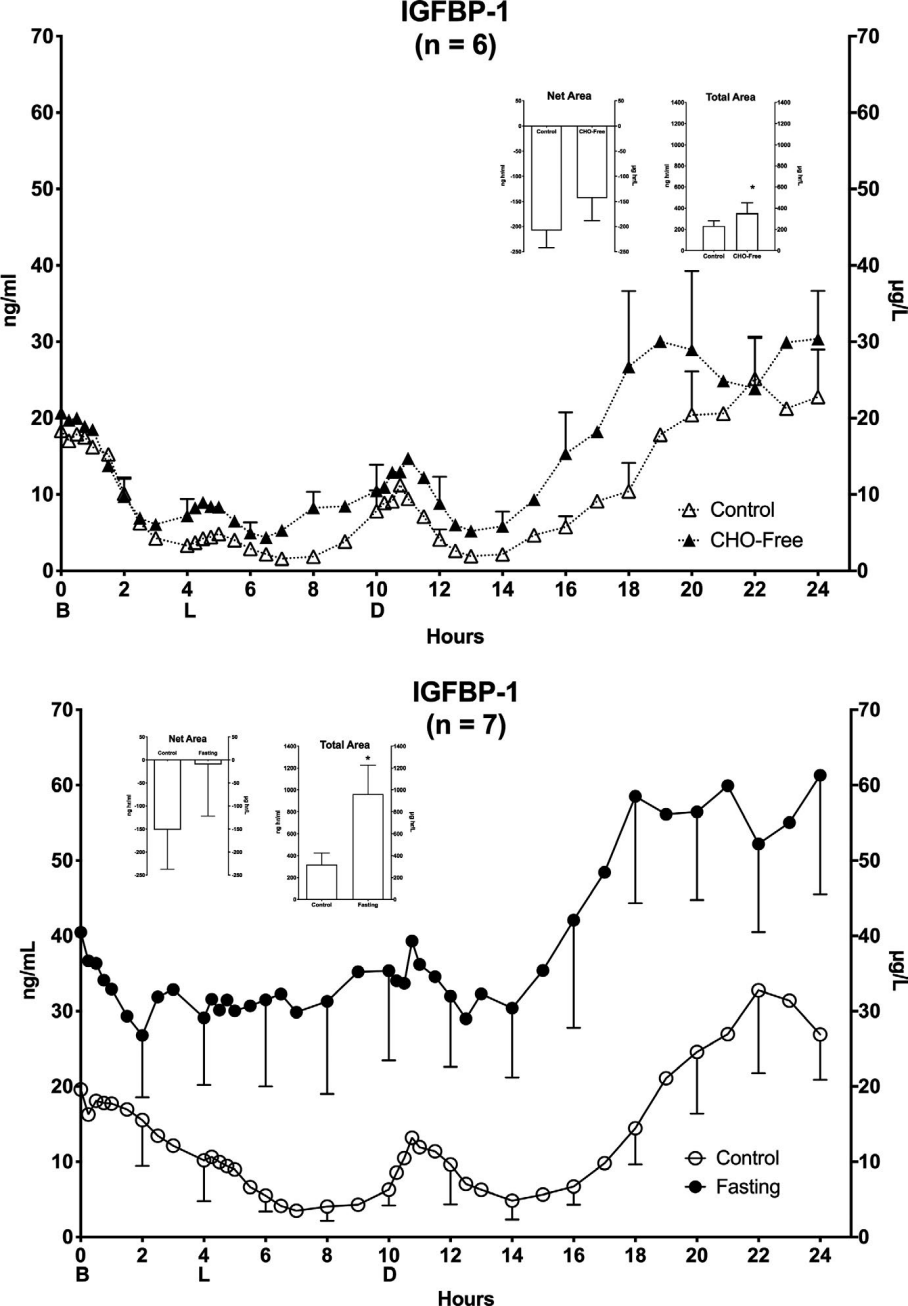
Serum Insulin‐Like Growth Factor Binding Protein‐1 (IGFBP‐1). Twenty‐four hour IGFBP‐1 responses. Top: (*n* = 6) Mean ± standard error of the mean (*SEM*) while ingesting the control diet (open triangles) and for the last 24 hr (hours 48–72) of ingesting CHO‐free diet (closed triangles). Bottom: (*n* = 7) Mean ± *SEM* while ingesting the control diet (open circles) and the last 24 hr (hours 48–72) of fasting (closed circles). X‐axis: Time in hours. 0 = 0800 hr. B, L, D indicate breakfast, lunch and dinner mealtimes. Y Axis: Left – concentration in Imperial units, Right ‐ concentration in Scientific Units. Inserts: Net Area represents the mean ± *SEM* integrated 24‐hr area response, using the overnight fasting concentration as baseline. Total Area represents the mean ± *SEM* integrated 24‐hr area response, using zero as baseline. S = Standard mixed diet (control), C = CHO‐free diet, F = Fasting. Statistics were done using Student's *t* test for paired variates. *p < .05

The mean IGFBP‐1 concentration at 0800 hr on the control diet was 18.3 ± 3.0 µg/L (Figure 4 – top). It decreased to 3.3 ± 0.7 µg/L at hr 4, increased very modestly and then decreased to a nadir of 1.9 ± 0.5 µg/L by hr 7. It subsequently increased modestly at hr 9, decreased again by hr 13 and then increased and was stable until the end of the 24‐hr period.

The CHO‐free diet resulted in the dynamics of the IGFBP‐1 response similar to those observed for the control diet. However, the concentrations were generally higher.

At the beginning of the control diet (day 1) for the fasting arm, the initial mean concentration was 19.6 ± 4.0 µg/L (Figure 4 – bottom), similar to the other control diet. The concentration decreased to a nadir of 3.51 ± 1.6 µg/L at hr 7, after which the dynamics also were similar to those observed during the other control diet.

On the last day of fasting, the initial mean IGFBP‐1 concentration at 0800 hr (day 4) was 40.5 ± 10.0 µg/L (2‐fold increase compared to the control diet) (*p* = .039). It was followed by a decline to 26.8 ± 8.2 µg/L at hr 2. The concentration was stable until hr 14 after which time it increased to 58.5 ± 14.2 µg/L and remained elevated for the duration of the 24‐hr period. Also, the final concentration remained elevated whereas, it was decreasing when the control diet was ingested.

Thus, with both a CHO‐free diet and with fasting, the IGFBP‐1 concentrations were lower during the day than at night. With fasting the diurnal variation was attenuated as expected.

#### 3.5.1 Areas

(Top insert) The 24‐hr integrated net IGFBP‐1 area responses (left) were −208 ± 34 and −143 ± 45 µg·hr/L for the control diet and the CHO‐free diet, respectively (*p* = .17). The total area responses (right) were 231 ± 50 and 354 ± 98 µg·hr/L for the control diet and the CHO‐free diet, respectively (*p* = .04).

(Bottom insert) The net IGFBP‐1 area responses (left) were −152 ± 85 and −10 ± 112 µg·hr/L, for the control and fasting, respectively (*p* = .21). The corresponding total area responses (right) were 318 ± 107 and 962 ± 263 µg·hr/L, respectively (*p* = .03), a 3‐fold increase. This was largely due to a higher initial concentration when the subjects were fasting.

### 3.6 IGF‐1 and IGFBP‐3

The overnight fasting‐free IGF‐I concentration (Table 1) decreased very modestly compared to the respective control concentrations. Only the decrease resulting from the CHO‐free diet was statistically significant (*p* = .01). The IGFBP‐3 concentrations remained essentially unchanged (Table 1).

### 3.7 Insulin

**FIGURE 5:**
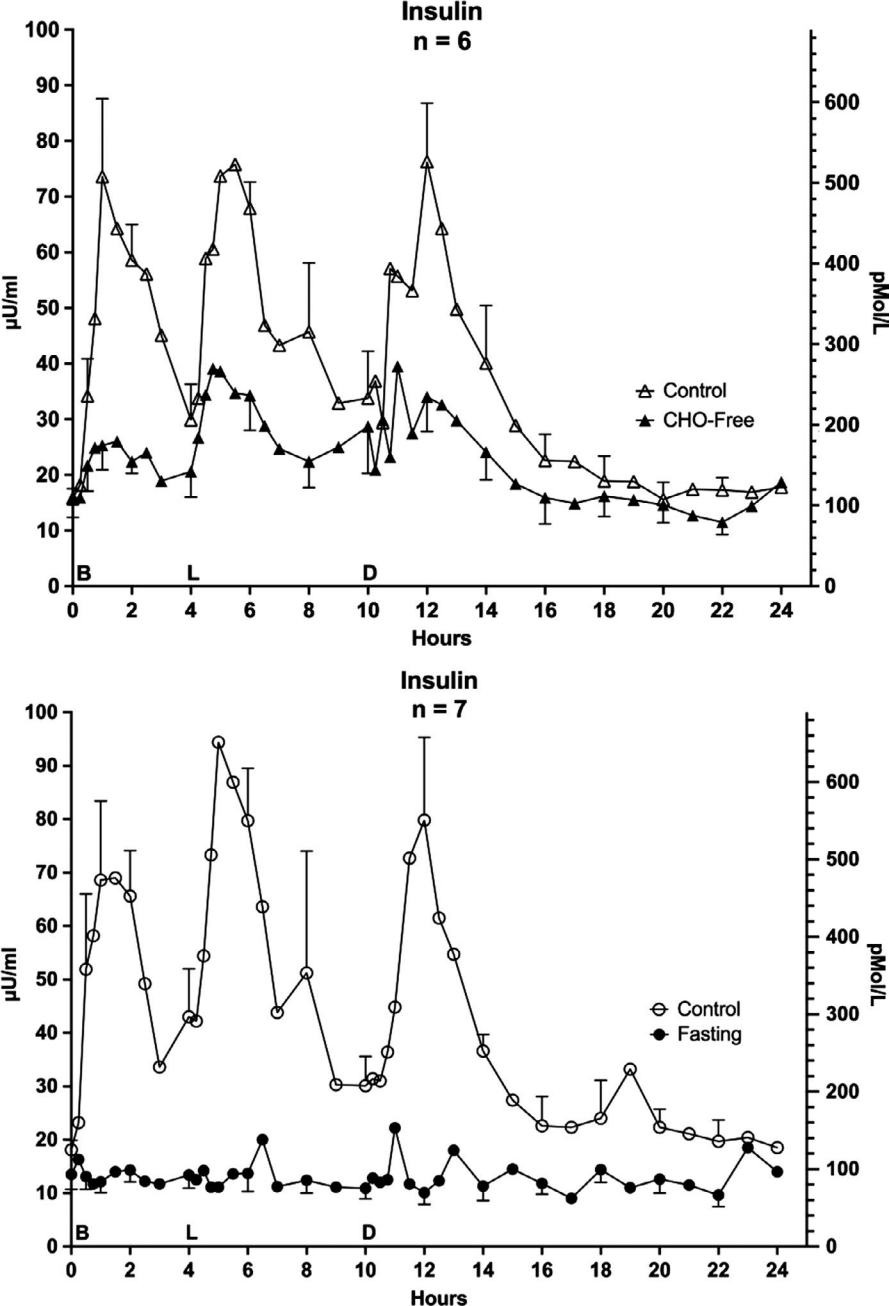
Serum Insulin. Twenty‐four hour profile for insulin responses. Top: (*n* = 6) Mean ± standard error of the mean (*SEM*) while ingesting the standard mixed diet (i.e. control – open triangles) and for the last 24 hr (hours 48–72) of ingesting a CHO‐free diet (closed triangles) Bottom: (*n* = 7) Mean ± *SEM* while ingesting the control diet (open circles) and the last 24 hr (hours 48–72) of fasting (closed circles) X‐axis: Time in hours. 0 = 0800 hr. B, L, D indicate breakfast, lunch and dinner mealtimes Y Axis: Left – concentration in Imperial units, Right ‐ concentration in Scientific Units. Redrawn from previously published data by Nuttall et al. (2015)

When on the control diet before the CHO‐free arm of the study, the mean insulin concentration at 0800 hr was 110 pmol/L (16 ± 2 µU/ml) (Figure 5 – top). It increased to a maximum after each of the meals, subsequently declined, but did not return to the overnight fasting concentration before the next meal. However, the concentration did return to the baseline (124 pmol/L or 18 µU/ml) the following morning.

The CHO‐free diet resulted in an increase in insulin concentration after each meal, similar to, but greatly attenuated compared to the control meals. The 0800‐hr concentration was identical to the control meals and returned to near baseline the following morning.

The dynamics of the insulin concentrations for the control before the fasting arm were very similar (Figure 5 bottom). At both the beginning and end of the last 24 hr of the fasting arm of the study the insulin concentration was modestly lower, 97 pMol/L (14 ± 3 µU/ml) and varied little during the day (Figure 5 bottom). At all but 2 time points the concentration was less than 140 pmol/L (<20 µU/ml).

The glucagon concentrations were not different between the diets or with fasting. They did not change significantly over the 24 hr, but decreased in the latter half of the 24‐hr period (See ref (Nuttall et al., 2015)).

### 3.8 Glucose

**FIGURE 6:**
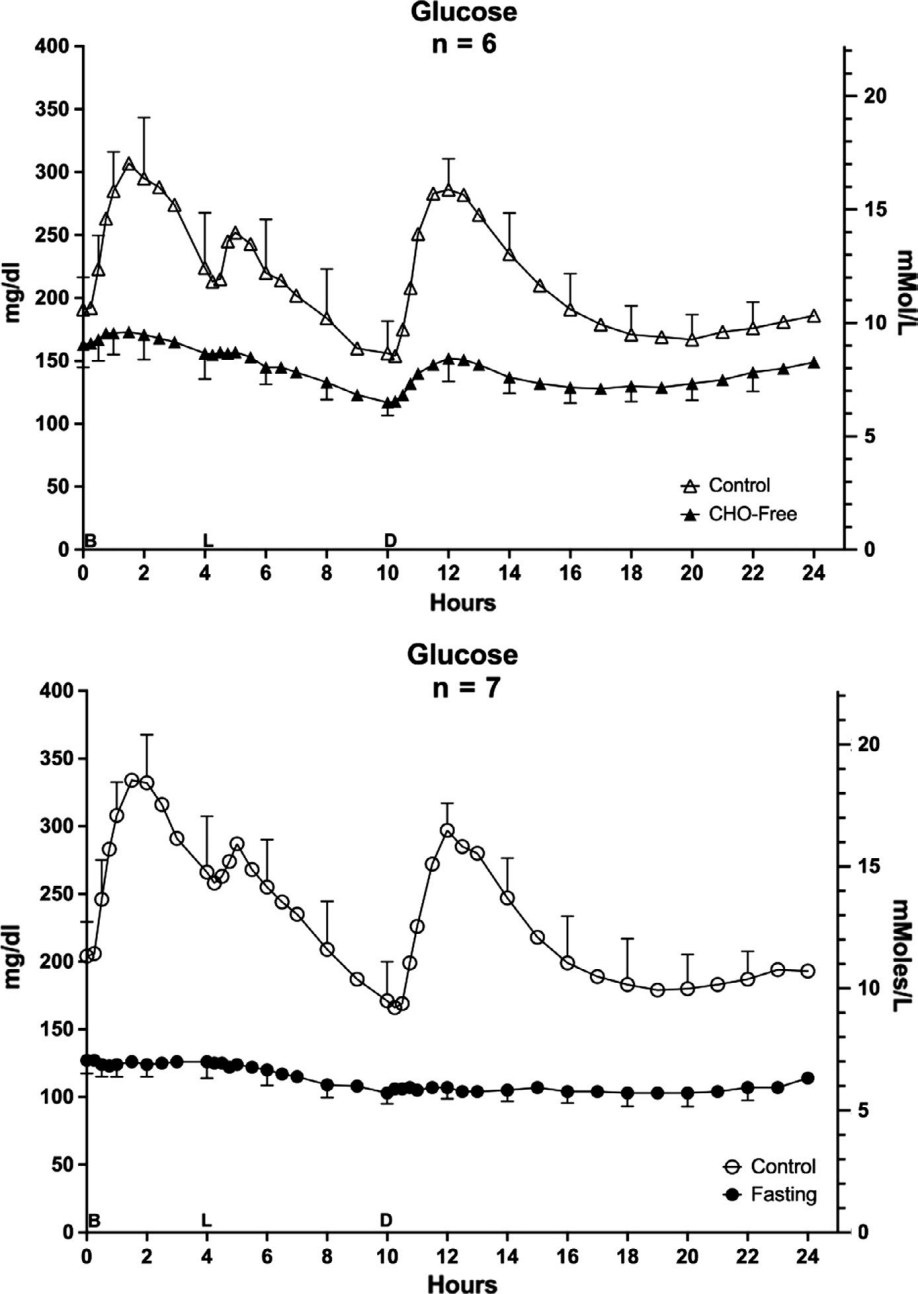
Serum Glucose. Twenty‐four hour profile for glucose responses. Top: (*n* = 6) Mean ± standard error of the mean (*SEM*) while ingesting the standard mixed diet (i.e. control – open triangles) and for the last 24 hr (hours 48–72) of ingesting a CHO‐free diet (closed triangles) Bottom: (*n* = 7) Mean ± *SEM* while ingesting the control diet (open circles) and the last 24 hr (hours 48–72) of fasting (closed circles)X‐axis: Time in hours. 0 = 0800 hr. B, L, D indicate breakfast, lunch and dinner mealtimes. Y Axis: Left – concentration in Imperial units, Right ‐ concentration in Scientific Units. Redrawn from previously published data by Nuttall et al. (2015)

When consuming the control diet before the CHO‐free arm of the study, the 0800 glucose concentration was 10.6 mmol/L (191 ± 26 mg/dl) (Figure 6 – top). It increased nearly immediately after each meal, and subsequently decreased before the next meal. The concentration was at the lowest point during the 24 hr just before the dinner meal (8.7 mmol/L or 156 ± 26 mg/dl). At the end of the 24 hr, it had returned to near the original overnight fasting concentration.

The CHO‐free diet resulted in a glucose profile similar to, but greatly attenuated compared to the control. The 24‐hr nadir was 6.5 mmol/L (117 ± 10 mg/dl).

The dynamics of the glucose concentrations for the control diet before the fasting arm was very similar (Figure 6 bottom). After 48 hr of fasting, the glucose concentration was 7.1 mmol/L (127 ± 10 mg/dl), It decreased to a low of 5.7 mmol/L (103 ± 10 mg/dl) at several time points during the last 24 hr with the final concentration at 6.3 mmol/L (114 ± 11 mg/dl). Thus, the glucose concentration was markedly attenuated with the CHO‐free diet compared to the control diet. With fasting the concentration was further decreased. Indeed, it was within the normal reference range during much of the final day of fasting. As with the other data, the reproducibility of the glucose and insulin data with the control diets were remarkable (Nuttall et al., 2015).

## 4 DISCUSSION

Our laboratory has had a long‐term interest in fuel metabolism and its regulation, both in normal subjects, but particularly in those with type 2 diabetes. A CHO‐free diet and fasting, the two interventions used in this study, allow comparisons of metabolic responses during the two most extreme examples of fat‐derived fuel, one exogenous, the other endogenous.

In normal subjects, the major signal for utilizing lipids for fuel is the reduced availability of glucose for oxidation, although insulin also is implicated, as elucidated in a series of elegant studies and reviews over the years by Klein, Wolfe, Sidossis, and other Associates (Klein, Sakurai, Romijn, & Carroll, 1993; Klein & Wolfe, 1992; Sidossis & Wolfe, 1996). Subsequently, Wolfe (1998) refined this concept to indicate it was the glucose metabolism rate that was controlling lipolysis. Others (Jensen, Caruso, Heiling, & Miles, 1989) reported that the rate of lipolysis is dependent on the insulin concentration in a postabsorptive state.

Therefore, in discussing the response of the circulating fat‐derived products (TAG, NEFA, bHB) in subjects with T2DM, the corresponding glucose and insulin concentrations also should be considered. To facilitate that discussion, the 24‐hr glucose and insulin profiles, previously published in a slightly different format (Nuttall et al., 2015) have be included herein (Figures 5 and 6).

Subjects with T2DM represent a large and highly variable cohort of individuals, all of whom have an elevated fasting glucose concentration and/or an abnormal post‐glucose challenge test. Commonly these subjects have normal or increased fasting insulin concentrations. The insulin response to post‐glucose testing also may be quantitatively normal or even excessive. However, subjects with T2DM nearly always have insulin resistance and an absent or impaired first‐phase insulin response to glucose testing (Fonseca, 2009; Rizza, 2010). Nevertheless, early in the time‐related progression of their metabolic dysfunctions, even with gross obesity, they can maintain essentially normal fuel metabolism, although at a higher glucose concentration (Fery, 1994). Also, the individuals generally are treatable without exogenous insulin.

Several factors affect postprandial plasma TAG responses in addition to the amount of fat in a meal. These include the rate of output of TAGs from the intestine, the rate of secretion of TAGs from the liver, the activity of lipoprotein lipases, etc. (Abumrad & Davidson, 2012; Cohn, McNamara, Cohn, Ordovas, & Schaefer, 1988). It is of interest that the TAGs reached a maximum ~1.5–2 hr after each meal, indicating a rather rapid and vigorous dose response, though not as rapid as the glucose and insulin responses.

In this study, the initial overnight fasting TAG concentration (Figure 1) was surprisingly similar whether the subjects ingested the control diet, the CHO‐free diet, or were fasting.

The overall net area TAG response to the CHO‐free diet was approximately 2‐fold greater than with the control diet (Figure 1 ‐ top). The dietary fat content was increased nearly 3‐fold, from 30% to 85% of total food energy. These data indicate a marked increase in clearance rate, such that the entire excessive dietary fat mass was completely cleared during the 24 hr.

With fasting, the TAGs were essentially unchanged throughout the 48‐ to 72‐hr period of fasting (Figure 1 – bottom). This indicates the increases observed when the control and CHO‐free diets were ingested were due to the food ingested and its composition only. Parenthetically, the disposal rate was such that whether merely potentially using ingested TAGs as a major fuel (fasting), or for storage (controls and CHO‐free diet), the initial and final concentrations were similar.

Clearly, in all three conditions, an adaptation was present which resulted in the rate of TAG clearance adjusting to the load requiring disposal, that is, storage and/or oxidation. Thus, the signal or integration of signals that resulted in this elegantly sensitive response is present in people with T2DM.

The current data also suggest the regulation of food‐derived fat processing apparently is by the mass ingested. That is, the data are compatible overall with mass action kinetics (i.e., the response equals the demand), and is not saturable under these extreme conditions.

In addition, the stability of the regulatory process of absorption, metabolism, and storage over time, as indicated by the similarity of the two mixed meal control diet data over the ~5 week time period between studies, is impressive (Figure 1 top, control versus Figure 1 bottom, control; also see Table 1).

Finally, in the present study for both diets (control and CHO‐free), the current quantitative TAG disposition data are not compatible with the concept of glucose control directly and/or indirectly. TAG disposition is not associated coordinately with the circulating glucose concentrations (Figure 6). The TAG disposition changes observed also were all being regulated efficiently, but at different glucose concentrations. When ingesting the control diet before the CHO‐free or fasting arms, the initial glucose concentrations were 10.6 and 11.3 mmol/L (191 and 204 mg/dl), respectively, (Figure 6). When ingesting the CHO‐free diet it was 8.9 mmol/L (160 mg/dl). With fasting it was 7.1 mmol/L (127 mg/dl). Also, the 24‐hr glucose net area responses were only positive when volunteers ingested the control diet (Nuttall et al., 2015).

Regardless of the greatly different plasma glucose concentrations, the overnight fasting (initial) insulin concentrations were very similar (2.7, 2.7 pmol/L (16, 16 µU/ml), control, CHO‐free diet and 3.0, 2.3 pmol/L (18, 14 µU/ml) control, fasting) (Figure 5). Thus, it is possible that the various glucose concentrations had been determined by the similar insulin concentrations, or vice versa. The 24‐hr insulin net area responses were positive when the volunteers ingested both the control diet and the CHO‐free diet, but slightly negative when fasting (Nuttall et al., 2015).

As pointed out in a seminal paper by Goodner, Conway, & Werbach (1969) over 50 years ago, the post absorptive glucose concentrations can be grossly elevated in people with T2DM, but the insulin concentrations are still within the normal reference range. Nevertheless, a prompt increase in insulin can occur when glucose is raised further, as demonstrated here. Also, the insulin concentration will remain further elevated until the glucose returns to the previous post absorptive glucose concentration although they all may be different. The mechanism that maintained the elevated postabsorptive glucose concentrations remained undetermined.

We are not aware of follow up of these observations in the literature. However, the current data are compatible with the observations by the above authors, that is the “fasting” post absorptive insulin and the meal stimulated insulin concentration are regulated individually. This we refer to as a resetting of the postabsorptive “glucostat” at a high stable level, if necessary, in order to maintain essentially normal fuel metabolism and a normal or modestly elevated insulin concentration. It is a term we prefer to that of glucose‐based “insulin resistance” (at least in reference to the postabsorptive glucose concentration). Nevertheless, the glucose/insulin meal responses are adequate to maintain a near normal disposition of ingested fuels but at a high stable postabsorptive glucose concentration.

It should be added that the 24‐hr insulin response in the present report was much smaller when the CHO‐free compared to the control diet was ingested (Figure 5) (Nuttall et al., 2015), even though the TAG clearance rate was greater than that observed when the mixed diet was ingested.

In summary, overall fat (TAG) metabolism is being regulated efficiently regardless of the great differences in glucose and insulin responses between the two diets. The post meal insulin responses (Figure 5) were those expected by the differences in insulin responses to the carbohydrate, protein and fat ingested in the control meals and to protein and fat in the CHO‐free meals, that is, the latter was independent of an exogenous source of glucose. In any regard, if insulin is playing a role in TAG metabolism its concentration was appropriate for complete storage and/or utilization of the respective TAGs ingested.

The NEFA concentration is negatively regulated by the circulating insulin concentration in normal subjects (Campbell, Carlson, Hill, & Nurjhan, 1992; Nurjhan, Campbell, Kennedy, Miles, & Gerich, 1986), in those with T2DM (Howard et al., 1979) and in those with T1DM as well (Jensen et al., 1989). Indeed, it is extremely sensitive. Even insulin variations within the usual normal overnight AM fasting range can influence its concentration (Jensen et al., 1989). Interestingly, insulin largely regulates the disposition of circulating NEFAs derived from the TAGs whether for storage or for oxidation. It also regulates the adipose cell lipolysis rate intracellularly when endogenous NEFAs are needed as a fuel (low insulin) or stimulates lipogenesis when incoming NEFAs need to be stored (high insulin). However, the bulk of NEFAs are released by adipose capillary‐associated lipases, which also apparently are insulin sensitive (Coppack, Jensen, & Miles, 1994).

In the current study, ingestion of the control meals resulted in a marked increase in insulin (Figure 5) (Nuttall et al., 2015). This was associated with a rapid and greatly decreased NEFA concentration except for a dramatic transient increase at hour 10, a time at which the insulin concentration was at a transient minimum (~ 5 pmol/L), although still twice as high when compared to the 0 hr (0800) concentration (Nuttall et al., 2015). This suggests the insulin sensitivity may be reduced, but if so, it was still sufficient to facilitate a rapid removal of NEFAs from the circulation. Indeed, it was sufficient to reduce the concentration far below the initial concentration (Figure 2). As noted in 1956 by Dole (1956), this occurred in normal subjects even though the TAG concentrations were increased, indicating NEFA clearance greatly exceeded the TAG entrance into the circulation.

When ingesting the CHO‐free diet (Figure 2 ‐ top), the initial 0 hr (0800) NEFA concentration was similar to that when subjects ingested the control meals. It then rapidly increased reaching a maximum at hr 12 that was far greater than when they ingested the control meals. It also more or less correlated with the large rise in TAG concentration. That is, the absorption and processing of the large amount of ingested fat and the resulting increase in TAG apparently provided a large substrate supply for NEFA release from adipose tissue capillaries and in excess of that which can be stored quickly in the adipose cells (referred to as a “spillover effect”) (Ruge et al., 2009). However, eventually the large amount of resulting NEFAs also were processed and resulted in a return to the initial concentration.

The response to the CHO‐free diet was associated with a considerably smaller, meal‐related insulin response compared to the control meals (Figure 5) (Nuttall et al., 2015), but apparently was still sufficient to clear all of the diet‐derived NEFAs, if indeed, the insulin concentration is the major regulator. Here the insulin rise likely was stimulated by the protein content of the meals and possibly to a minor degree by the high TAG concentration itself (Cahill et al., 1966; Manco et al., 2004; Nuttall, Mooradian, Gannon, Billington, & Krezowski, 1984). We cannot explain the sudden increase at the last time point and doubt that it was the beginning of an elevated NEFA concentration over the next 24 hr. However, it is a possibility.

Forty‐eight hours of fasting resulted in a ~55% increase in the 0 hr (0800) NEFA concentration. The concentration then remained rather stable until the 23rd hr when it increased (Figure 2). In normal subjects, short‐term fasting also results in a similar rise in the AM NEFA concentration (Cahill et al., 1966). This has been attributed, at least in part, to a decrease in the insulin concentration, which in turn is due to a decreased glucose concentration (Cahill et al., 1966) and utilization rate (Sidossis & Wolfe, 1996).

In this study, the sustained increase in NEFAs during fasting was associated with an unchanged 24‐hr insulin concentration (Figure 5) (Nuttall et al., 2015) and TAG concentration (Figure 1). Thus, the stably elevated NEFA concentration was ~2‐fold greater than when the men ingested either of the other 2 diets even though the initial glucose concentration was decreased, however not into the normal range. The mechanism that explains the elevation is not apparent. The insulin and TAG concentrations were unchanged. It could be explained by an increase in catecholamines (Carlson, Snead, & Campbell, 1994; Li & Sun, 2018), and/or peripheral or central nervous system input (Goodner, Tustison, Davidson, Chu, & Conway, 1967). These were not determined. It should be noted that the AM insulin concentration was similar to that when the men were being fed and in whom the glucose concentrations were much higher.

Mirani‐Oostdijk and associates (Mirani‐Oostdijk, van Gent, Terpstra, Hessel, & Frolich, 1981) reported similar 24‐hr NEFA profiles in three men with type IV hypertriglyceridemia after 2 weeks on a 65% CHO and subsequently on a 65% fat diet for 2 weeks. In the study by Goodner et al. (1969), the NEFA concentration was not within the normal reference range, unlike in the present study. The authors (Goodner et al., 1969) speculated that the elevated glucose concentration but normal insulin concentration was a glucose‐based mechanism for assuring normal amounts of NEFA for fuel, etc. in spite of the high glucose concentrations, that is, this could be expanded to include all other insulin‐regulated processes. The current data are compatible with this concept.

The “ketone bodies” consist of three compounds, acetoacetate (AcAc), β‐hydroxybutyrate (bHB), and acetone. AcAc is the original compound synthesized in vivo and is the form utilized directly as fuel. bHB is the reduced form of AcAc and circulates in the highest concentration. bHB is converted to AcAc before being utilized as a fuel. Acetone is generated in the lungs and is released in the expired air. Not only is it volatile, but it is also soluble in both water and liquid lipids. It is not a major fuel but can be converted into glucose (Puchalska & Crawford, 2017). Generally, all three forms change in concentration together in various metabolic states. bHB usually is present in the highest concentration and is assayed as a representative of ketone body concentrations and utilization in general, as was done in the present study. The ketone bodies are derived from NEFAs, which in turn are products of TAG de‐esterification. The circulating ketone body concentration, just as with the NEFAs, also is considered to be largely dependent on the availability of insulin (Soeters et al., 2009). The latter strongly inhibits AcAc production in the liver (Alberti, Johnston, Gill, Barnes, & Orskov, 1978; Soeters et al., 2009), but also increases both AcAc and bHB clearance rate (Keller, Gerber, & Stauffacher, 1988).

As indicated above, ketone body production is very sensitive to changes in insulin concentration. When insulin secretion is absent, impaired, or just down regulated, such as with prolonged fasting, increases in keto acids of approximately 100 fold are obtained routinely (Cahill et al., 1966). In the present 3‐day study, the fasting bHB concentrations were increased ~20‐fold. They also can be grossly elevated even when the NEFA concentration is not elevated out of the normal reference range (Cahill et al., 1966).

In normal humans, the liver can produce up to 300 g of total ketone bodies daily, and in the fed, fasted, and prolonged starvation state can contribute between 5% and 20% of the total daily energy requirements (Puchalska & Crawford, 2017). Thus, even in the fed state they are a significant contributor to the fuel supply.

In people with T2DM, the circulating post‐absorptive ketone body concentrations generally are normal or only modestly elevated. However, they may be elevated when the diabetes is being treated with Sodium‐Glucose Transporter‐2 (SGLT2) inhibitors (Polidori et al., 2018).

In this study, when these volunteers ingested the control meals, bHB was measurable but within normal limits throughout the 24‐hr period. It exhibited further episodic increases at times when the NEFA concentration was the highest (Figure 2) and the insulin concentration was lowest (Figure 5) (Nuttall et al., 2015).

Also, when ingesting the control meals, the bHB data were essentially identical at the two times when they were done weeks apart. As indicated previously, the postabsorptive insulin concentrations in general were only modestly higher than in lean, normal subjects and a rapid, major increase in insulin occurred with each meal (Figure 5) (Nuttall et al., 2015). The final mean concentration was similar to the initial concentration (Nuttall et al., 2015).

With the CHO‐free diet, there was a major increase in the overnight AM post absorptive bHB concentration compared to the control diet (297 µmol/L vs. 64 µmol/L), as expected, although the initial insulin concentrations were identical. This is difficult to explain if insulin is the major regulator of ketone body metabolism. (Catecholamines were not determined.) The 24‐hr profile also was hugely elevated (Figure 3 – top). Nevertheless, the concentration had returned to near the initial concentration by 24 hr.

The CHO‐free diet resulted in only a modest daylong increase in insulin and had returned to the previous AM concentration (Figure 5 – top) (Nuttall et al., 2015). Thus, this response likely represents a new 24‐hr steady state when compared with the normal diet.

Unless similar effectors are all involved in the regulation of TAG, NEFA, and bHB concentrations, each of which return to or near their initial value, it is impressive how the independent regulators are integrated regardless of the type of meals ingested.

When these men were fasting, the overnight AM concentration of insulin was similar to that observed when ingesting the control meals (Figure 5 – bottom). It remained stable during the subsequent 24 hr of observed fasting. The overnight fasting bHB concentration was grossly elevated and continued to increase although the insulin concentration remained unchanged, as did the NEFA concentration (Figure 2 – bottom), a substrate for ketone body production. Clearly neither was regulating the ketone body production in order to eventually establish a new homeostatic state, unless there is a daily progressive decreased feedback mechanism for ketone body concentration. Long‐term homeostasis requires ~17 days of fasting (Cahill, 1976; Owen, Felig, Morgan, Wahren, & Cahill, 1969).

The mechanism or mechanisms that regulate these observed data, that is, the regulation of bHB production and clearance rates, were not addressed in the present report. Clearly insulin plays a role and is grossly inversely concordant with the bHB concentrations. Other effectors undoubtedly are present, including mechanisms that rapidly respond to the overall fuel requirements for the entire body such as catecholamines, cortisol or CNS input, and not determined here.

The NEFAs had a dynamic change similar to that of the bHB. Since NEFAs are the precursors of the bHB, the changes in bHB could potentially be explained, at least in part, by the NEFA concentration changes. Also, as indicated, both are reported to be highly regulated by insulin. However, the concentration dynamics of the bHB were different from that of insulin (Figures 2 and 5). The current data indicate that ketone body metabolic regulation in men with T2DM when not fasting, just as with the TAG and NEFA concentrations, is sufficient to maintain a stable bHB concentration in the postabsorptive state.

In summary, at least in these men with type 2 diabetes, the metabolic responses to the changes in diet could represent a re‐setting of the glucostat at different defined high levels in order to maintain a normal insulin concentration and near normal fuel metabolism, but also at an elevated ketone body concentration. Nevertheless, the role of glucose/insulin in regulation of these fat‐derived fuels remains to be better defined. It also is apparent (Figure 3 – bottom) that fasting results in a much greater increase in bHB than does merely removing carbohydrate from the diet, at least over this time frame. Effectors other than insulin undoubtedly are very important.

Overall, the data suggest that these men with currently untreated T2DM have relatively normal fat metabolism at least in response to a normal mixed diet, a CHO‐free diet, and with short‐term fasting. They are functioning at a higher than normal glucose concentration, but only a modestly increased insulin concentration.

Insulin and IGF‐I are known to regulate protein metabolism, particularly in skeletal muscle. Insulin largely regulates the general rate of protein degradation (Liu & Barrett, 2002). IGF‐I is a major stimulator of protein synthesis (Russell‐Jones et al., 1994); it also has numerous other effects not reviewed here (Rajpathak et al., 2009). IGF‐I is known to be regulated by growth hormone and also by the protein content of the diet (Gannon & Nuttall, 2011). Both increase the circulating concentration and presumably the production rate. (See (Nuttall, Gannon, Saeed, Jordan, & Hoover, 2003) for further details).

In this study, only the overnight fasting (0800 hr) IGF‐I concentrations were determined. However, we (Gannon & Nuttall, 2011), and others (Rajpathak et al., 2009) have previously reported that Free IGF‐I concentrations are stable over 24 hr. Thus, the current hour 0800 results likely reflect the IGF‐I concentration throughout each 24‐hr period.

When compared to the control diets, there was only a decrease of 12% when the CHO‐free diet was ingested, that is, a partial adaptation to fasting, and a decrease of only 6% with fasting (Table 1). Thus, the small decreases in the present subjects with diabetes were considerably less than in normal subjects (> 45%) (Clemmons et al., 1981; Isley, Underwood, & Clemmons, 1983; Norrelund et al., 2003). The reason for the difference is not apparent to us. In normal subjects, fasting for 5 days has been reported to lower the overnight AM fasting IGF‐I concentration by ~60% (Isley et al., 1983).

Unlike the reported constant free IGF‐I concentration, the IGFBP‐1 demonstrated a dynamic circadian rhythm with a lower concentration during the day but elevated during the night when the subjects fasted. It is the only one of the six well‐characterized mammalian IGF‐I‐binding proteins to do so. However, the rhythm occurred at a concentration approximately twice normal. In addition, an inverse relationship between IGFBP‐1 and insulin has been reported in young as well as in aged subjects (Reviewed in (Gannon & Nuttall, 2011)). In a previous study, we also reported a 24‐hr inverse relationship between IGFBP‐1 and insulin in subjects with T2DM ingesting a control diet (Gannon & Nuttall, 2011). The data also suggested a rise in insulin concentration to 5.0–6.7 pmol/L (30–40 µU/ml) before a decrease in IGFBP‐1 concentration was observed.

It is of interest that the control diets were calculated to be the same as in our previous study (Gannon & Nuttall, 2011) and the IGFBP‐1 and insulin concentrations also are very similar, although the subjects in that study were all different volunteers. In this study, the changes during 24 hr also were similar when the subjects ingested the control diet on two different occasions, indicating the reproducibility of the data.

When the CHO‐free diet was ingested (Figure 4 – top), the IGFBP‐1 concentration was modestly increased compared to that when the control diet was ingested. This occurred although the insulin concentration changes were greatly different, possibly suggesting a high sensitivity to insulin changes or independence from insulin when fasting (Figure 5). With fasting the overnight (0800 hr) mean concentration had increased 2‐fold (Figure 4 – bottom). However, a circadian rhythm was present. The insulin concentration was decreased and remained stable during the 24 hr. This suggests that the circulating changes are not insulin regulated. At least it suggests that circadian regulators independent of insulin also are playing an important role in these men with T2DM.

The postabsorptive total cholesterol, LDL‐, HDL‐, VLDL‐, Non‐HDL‐cholesterol, and IGFBP‐3 data were essentially unchanged (Table 1).

### 4.1 Strengths and weaknesses

This is a well‐controlled observational study to determine the metabolic response to a predominantly exogenous fat‐fuel supply (CHO‐free diet) and with an endogenous fat‐fuel supply (fasting). The same subjects participated in both arms of the study, and each arm had its own standard mixed meal diet control. Meals contained commonly available foods, and the diets consisted of carefully designed macronutrient compositions. Complete 24‐hr profiles with numerous data points were obtained before and at the end of the study for TAGs, NEFAs, bHB, and IGFBP‐1.

The limitations of the study are: Only seven men with T2DM were studied; women were not studied. Also, the amount of caffeine consumed, if any, was not quantified. The issue of caffeine effects on fat and glucose metabolism is complex. However, it appears to have little or no effect on fat and glucose metabolism when ingested in usual amounts. Nevertheless, since this was not monitored, it possibly could have modestly affected the results obtained. This is a potential variable that was not controlled. In addition, a potential criticism may be the short duration (72 hr) of the study. However, this is a time over which the metabolic effects of fasting are apparent, and weight loss, and a change in body composition have been reported to be absent. Thus, a conclusion can be drawn on effects of endogenous versus exogenous fat fuel per se, without major structural body changes. The time frame we do not consider to be a weakness.

## 5 CONCLUSION

The 24‐hr response profiles for TAG, NEFA, bHB, and IGFBP‐1 were obtained when ingesting a mixed control diet before, and during the last 48–72 hr when ingesting a CHO‐free diet or fasting. A randomized cross‐over design was used. Total cholesterol, LDL‐cholesterol, HDL‐cholesterol, VLDL‐cholesterol, Non‐HDL‐cholesterol, IGF‐1, and IGFBP‐3 also were obtained. The 24‐hr profiles for insulin, glucose, and glucagon were obtained previously (Nuttall et al., 2015).

The glucose and insulin concentrations as well as the TAG, NEFA, and bHB concentrations all had essentially returned to the initial concentration at the end of the last 24‐hr period, that is, by the next morning, thus maintaining overall fuel homeostasis, although at higher than normal postprandial and 24‐hr integrated glucose concentrations produced in each protocol. Only fasting was not sufficiently long for ketone bodies to reach homeostasis. The TAGs and NEFAs had attained 24‐hr homeostasis, as had the glucose and insulin concentrations, that is, all had essentially returned to the initial concentration present when subjects ingested the control diets.

These data are compatible with two different insulin regulatory systems, one for determining the postprandial insulin concentration regardless of the glucose concentration and one regulating the response to food ingestion. They are largely independent. Also, the postprandial concentration is at least partly glucose independent.

The current data indicate that male patients with type 2 diabetes, in the absence of glucose‐lowering pharmaceutical agents are still able to absorb, process, utilize, and if necessary, store large amounts of ingested fats adequately, as well as adjust adequately to short‐term starvation. However, this occurs in the presence of very high circulating glucose concentrations.

These data are largely observational. Future investigations are required to determine the adjustments in metabolic pathways and control points resulting from these dietary changes.

As pointed out by Harber et al in 2005 (Harber, Schenk, Barkan, & Horowitz, 2005) “Little is known about the metabolic effects of a CHO restriction without the confounding influence of weight loss or negative energy balance.” The information available since has improved our understanding of the metabolic effects. However, much remains to be learned.

## CONFLICT OF INTEREST

The authors declare that they have no competing interests.

## AUTHORS’ CONTRIBUTIONS

Dr. R. M. Almokayyad was an Endocrine Fellow in training at the time this study was done. Dr. Almokayyad applied for and obtained IRB approval for the study, recruited the subjects, obtained the blood specimens, performed the majority of the indirect calorimetry, and contributed to the data analysis. Drs. F.Q. Nuttall and M.C. Gannon obtained the funding, formulated and designed the study, performed the final analysis of the data and wrote the final manuscript. All authors have read the final manuscript, agreed to its content and approved the final manuscript. All authors are accountable for the accuracy and integrity of the data.

## DISCLAIMER

The contents of this manuscript do not represent the views of the United States Department of Veterans Affairs or the United States Government. The Department of Veterans Affairs had no involvement in the study design, the collection, analysis and interpretation of data, in the writing of the manuscript or in the decision to submit the manuscript for publication. The authors declare no conflicts of interest.

## Data Availability

Following the current regulations of the US Government, final data sets underlying publications resulting from the research will not be shared outside the VA except as required under the Freedom of information Act (FOIA).
